# Contemporary Surgical Management of Deep-Seated Metastatic Brain Tumors Using Minimally Invasive Approaches

**DOI:** 10.3389/fonc.2018.00558

**Published:** 2018-11-28

**Authors:** Lina Marenco-Hillembrand, Keila Alvarado-Estrada, Kaisorn L. Chaichana

**Affiliations:** Department of Neurosurgery, Mayo Clinic, Jacksonville, FL, United States

**Keywords:** brain metastases, laser, LITT, minimally invasive, tubular retractors

## Abstract

A subset of metastatic brain tumors occurs in deep-seated locations. Accessing and resecting these lesions can be associated with significant morbidity because it involves large craniotomies, extensive white matter dissection, prolonged retraction, and risk of inadvertent tissue injury. As a result, only palliative treatment options are typically offered for these lesions including observation, needle biopsies, and/or radiation therapy. With the development of new surgical tools and techniques, minimally invasive techniques have allowed for the treatment of these lesions previously associated with significant morbidity. These minimally invasive techniques include laser interstitial thermal therapy and channel-based resections.

## Introduction

Metastatic brain cancer (MBC) is the most common type of brain tumor in adults (1, 2). It is estimated that there will be more than 200,000 new cases each year in the United States alone (1, 2). The most common sources are the lung, breast, kidney, colon, and skin, where approximately 20–30% of patients with these primary cancers will develop a brain metastasis (1, 2). The treatment of primary cancers has improved; however, the ability to prevent MBC and prolong survival for patients who develop MBC has not (1, 2). The treatment options for patients with MBC include some combination of surgical resection, radiation therapy, and/or chemotherapy (1, 2). The goals of these therapies are to primarily prevent local tumor progression (3– 6).

The majority of brain metastases occur at the gray-white junction (7, 8) These metastatic cancers are thought to breach the blood-brain barrier in areas of slow flow, which is typically in watershed regions and the ends of small perforating vessels (7, 8). As a result, most of these lesions are cortically based or in close juxtaposition to the cerebral cortex and/or cerebellar hemisphere (7, 8). When surgery is pursued for these typical lesions, the distance of brain parenchyma that must be traversed is relatively short (3–6). However, some metastases can occur in deep-seated, eloquent regions such as the thalamus, basal ganglia, and deep cerebellar nuclei (7, 8). When these deep-seated lesions occur, patients are typically symptomatic from mass effect and eloquent nuclei and white matter tract (WMT) involvement, and surgical treatment is more challenging because of the morbidity associated with accessing and resecting these lesions (9–12). In this review, we will discuss the use of contemporary surgical management of these lesions using minimally invasive approaches, namely laser interstitial thermal therapy (LITT) and channel-based resections (9–12).

## Surgical indications for brain metastases

Patients who present with MBC can undergo various treatments including surgical resection, radiation therapy, and/or chemotherapy (1, 2, 13–17). The choice of therapies is typically predicated by an estimation of a patient's prognosis, where generally more localized (surgery, stereotactic radiosurgery) and aggressive therapies are offered to patient's with better prognoses (3, 18). In order to predict survival, there are several prognostic scoring systems that have been developed including the Recursive Partitioning Analysis (RPA), Score Index For Radiosurgery (SIR), Basic Score for Brain Metastases (BSBM), Rotterdam system (ROTTERDAM), Golden Grading System (GGS), Rades classification (RADES), and Graded Prognostic Assessment (GPA) classification systems.

In general, surgery for brain metastases are indicated for patients who possess good prognoses and accessible lesions with low potential associated morbidity (3–6). However, surgery is often pursued for large lesions (< 3 cm), lesions with significant mass effect, and/or symptomatic lesions, even for palliative purposes (3–6). Lesions that are large and deep-seated, however, represent a surgical dilemma (10–12). For metastatic lesions that are small with minimal edema and mass effect, radiation therapy, namely stereotactic radiosurgery, is preferred (10–12). This is because historically accessing and resecting lesions has been associated with significant surgical morbidity (10–12). This morbidity is associated with accessing, visualizing, resecting, and achieving hemostasis (10–12). Deep-seated tumors have typically required large craniotomies and large dural openings to accommodate bladed retractor systems (10–12). These bladed retractor systems require a large footprint in order to be effective (10–12). In addition, the superficial cortex and overlying white matter have to be retracted to provide exposure of the underlying lesion (10–12). These retractor blades can induce significant damage by retractor-applied sheer forces, especially when multiple retractors are used, ischemia from contact pressure under the retractor blades, and potential tissue injury when left unprotected between the blades during repeated accessing the lesion with surgical instruments (10–12). As a result, offering surgery for deep-seated brain tumors has been limited. However, some deep-seated metastatic brain tumors are symptomatic and can have significant mass effect including hydrocephalus (10–12). In these cases, surgery is warranted because of the delayed effect of non-surgical options such as radiation therapy. There are, however, no clinical trials that specifically address surgery for deep-seated metastatic tumor, as they represent a smaller subset of metastatic tumors. The use of minimally invasive technique including LITT and channel-based retraction, however, have allowed for a potentially safer surgical options for these lesions (10–12).

## Laser interstitial therapy (LITT)

LITT is a minimally invasive technique that was initially used in the 1980s, and used to treat difficult to access lesions including malignant gliomas, radiation-resistant metastases, epileptic foci, and radiation necrosis (19–22). This involves making a burrhole over the intended trajectory, insertion of a skull bolt, and placement of a probe affixed with an optical fiber into the lesion through the bolt under stereotactic navigation (19–22). The optical fiber is used to heat the surrounding tissue causing coagulative necrosis, with the goal of sharp drop off in temperature effects to minimize damaging the surrounding peri-lesional tissue (19–22). The thermal effects of the interstitial laser can be measured with MR thermometry and cooled with carbon dioxide or saline (19–22). The lesion itself can enlarge from edema associated with cell swelling and necrosis from the thermal effects up to 1.5–5 times its original size and be enlarged for up to 40 days until there is resorption of the necrotic center (19–22). The resorption can take over 6 months (19–22). The advantages of LITT as opposed to standard craniotomies include smaller incision, less blood loss, less parenchymal manipulation, shorter hospital stay, and ability to perform adjuvant therapies sooner because of the lack of need for incisional healing with smaller incisions (19–22). The disadvantages include difficulty with treating large lesions, lesions with significant edema, and highly vascular lesions (19–22). The biggest concern is the transient volume increases in the immediate postoperative period that can lead to increased mass effect and neurological deficits, necessitating pharmacotherapy or surgical therapy (19–22).

There are two principle companies that provide LITT are Monteris^TM^ (Neuroblate® and Medtronic^TM^ (Visualase®) (19–22). The Neuroblate® system uses a CO_2_ gas-cooled laser probe and has both side-firing and diffuse-tip laser applications (19–22). Similar, but different, the Visualase® system uses a diode laser generator and has a cooling catheter than contains a 1-cm-long fiberoptic applicator with a light-diffusing tip, where the catheter is connected to a peristaltic roller pump that circulates sterile saline to cool the probe tip and surrounding tissue (19–22). It also provides thermal delivery in an ellipsoid-cylindrical pattern (19–22). Both systems are connected to an MRI unit and computer workstation that allows robotic manipulation and real-time thermographic data, where predetermined peri-lesional thresholds can be pre-assigned (19–22).

The majority of studies on the use of LITT for metastatic brain tumors are small institutional series with < 10 patients (19–22). Carpentier et al. reported the use of LITT in 7 patients with 15 metastatic lung and breast adenocarcinomas with lesion sizes ranging from 1 to 3 cm in diameter of unknown locations (19). All patients were discharged within 24 h, had no new deficits, and the median survival was 19.8 months (19). Hawasli and colleagues reported their institutional series of 17 LITT cases, where five had brain metastases and prior therapy including surgery and radiation therapy (21). The lesions ranged from 5.2 to 9.9 cm^3^ and involved the WMT of the frontal, parietal, frontoparietal lobes and the insula (21). Two of the five patients had transient deficits including aphasia and hemiparesis (21). The median progression free and overall survival of these patients was 5.8 months (21). Eichberg et al. documented the use of LITT in four patients with recurrent cerebellar metastases, where the sizes ranged from 1.1 to 7.2 cm^3^ and the postop volume ranged from 0.5 to 7.6 cm^3^, where lesion size increased by an average of 487% on postoperative day 1 and the time it took to shrink below initial volume was 295 days (20).

LITT is typically reserved for metastatic brain tumors that have failed radiation therapy (19–22). It provides a minimally invasive way to target both deep-seated and superficial metastatic lesions that have not responded to radiation therapy (19–22). Its use, however, is tempered by the transient increase in tumor volume that can persist for months (19–22). Therefore, the use of LITT is not typically used as the initial treatment of metastatic brain tumors and for lesions with significant mass effect and/or in close proximity to eloquent structures (19–22). Interestingly, in a recent study by Sloan and colleagues, they reported the use of LITT followed by transportal resection in 10 patients with brain tumors (1 MBC) (23). This use may expand the use of LITT therapy for MBC (23).

## Channel-based resections

Tubular or channel-based retractors provide a means to access deep-seated lesions (9–12). The typical approach to deep-seated lesions involved large craniotomies, sizeable cortisectomies, extensive white matter dissections, and use of multiple bladed retractors to create a large enough corridor to provide visualization, access, and resection (24). This approach is associated with potential injury as a result of large exposures, prolonged retraction, and inadvertent tissue injury during access and resection (24). Channel-based retractors circumvent a lot of these limitations (9–12). In this approach, a circular channel is placed into the brain typically through a sulcus (9–12). This channel displaces rather than severs the WMT, provides a protected corridor for accessing and resecting the lesion, and creates equivalent, circumferential radial forces to minimize collateral injury (9–12). These retractors were first used in the 1980s, and their use has expanded to intracranial hemorrhages, gliomas, vascular lesions, and MBC, among others (9–12).

The most widely used channel-based retractors are peel-away catheters, oval-shaped retractors, and circular retractors (9–12). The peel-away catheters (Medtronic^TM^) are similar to central line peel-away catheters whose diameters are typically measured in French (9–12). These catheters are typically limited to ventricular surgery as they require working channel endoscopes for visualization and resection and a clear fluid medium (9–12). The advantages are they are the least invasive, can be used through burrholes, and the least disruptive for white matter tracts (9–12). The disadvantages are they are limited to clear fluid media, obviate bimanual techniques because require working-channel endoscopes, and hemostasis can be challenging (9–12). Oval-shaped retractors (Viewsite Brain Access System®, Vycor^TM^) comes in a variety of lengths (30–70 mm) and widths (12–28 mm). The oval-shaped retractors can be applied to both deep-seated ventricular and parenchymal lesions (9–12). The advantages of oval-shaped retractors are they allow bimanual techniques and have greater widths for maneuverability, but the disadvantages are that they have inequivalent radial retraction because of the oval shape, can severe white matter tracts at wider widths, and are difficult to use through sulci because of the blunt tip (9–12). Circular shaped retractors (Brainpath®, Nico^TM^) also come in a variety of lengths (50–95 mm) and widths (11.5–13.5 mm) and can also be applied to both deep-seated ventricular and parenchymal lesions (9–12). The advantages of circular retractors are they provide equivalent radial retraction, can be applied to the sulcal space, and allow bimanual techniques (9–12). The primary disadvantage of the circular retractors is they are narrower than the oval-shaped retractors with less maneuverability (9–12).

**Figure 1:**
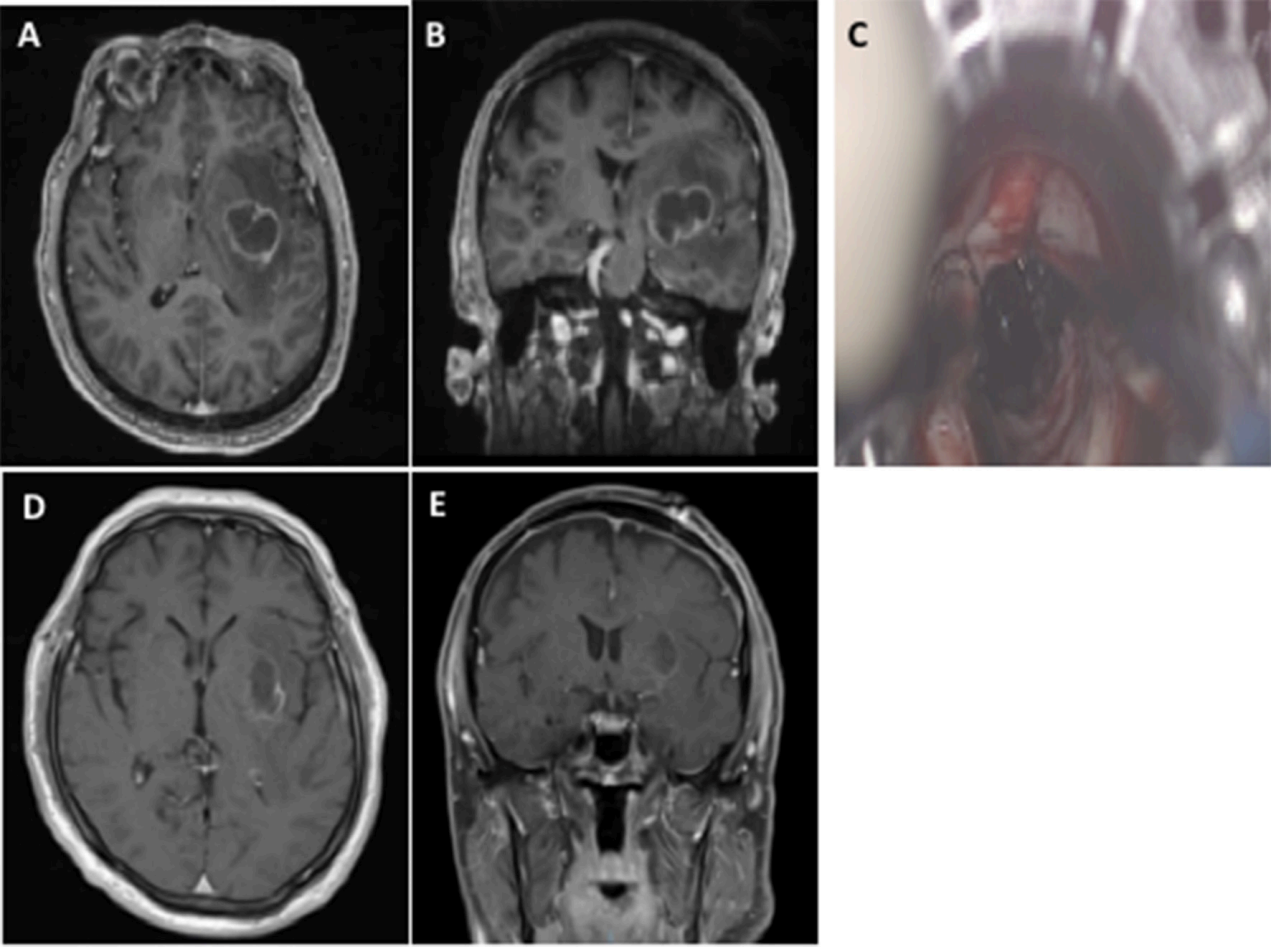
The use of channel-based retractor of a left basal ganglia non-small cell lung cancer brain metastasis. Preoperative axial **(A)** and coronal **(B)** MRI with contrast demonstrating a deep-seated left basal ganglia brain metastasis. The use of a channel-based retractor to access the lesion **(C)**. Postoperative axial **(D)** and coronal **(E)** MRI with contrast demonstrating gross total resection and no superficial cortical and white matter changes.

There are an expanding number of case series that have evaluated the use of these channel-based retractors for MBC (Figure 1) (9–12). Bakhsheshian et al. performed a multi-center study with 25 patients with metastatic brain tumors, where gross total resection was achieved in 80%, 1 (4%) had a new neurological deficit, and 19 (76%) had improved neurological symtpoms (9). These lesions were frontal (*n* = 5), parietal (*n* = 8), cerebellar (*n* = 8), occipital (*n* = 3), and splenium (*n* = 1) (9). Day reported a single surgeon experience with this approach in 20 metastatic brain tumors, where gross total resection was achieved in 19 (95%), postoperative hemorrhage in 1 (5%) that did not require evacuation, new deficit in 0, and perioperative mortality in 1 (5%) due to pulmonary complications (25). More recently, we reported our experience in 50 consecutive channel-based resection cases, where 14 had brain metastases (10). All of these patients underwent gross total resection and no patients had worsening neurological deficits (10).

Channel-based retractors allow a protected corridor for accessing and resecting deep-seated brain metastases that are at least below the deepest sulcal boundary (10–12). It provides a minimally invasive ability to access these lesions that previously were not resected, offered only needle biopsies, or offered surgery with significant risks (10–12). The tubular retractors, however, are narrow (approximately 13.5 mm in diameter), making it difficult to maneuver, establish hemostasis, and visualize feeding vessels (10–12). This narrow corridor also obviates certain instruments that are wide in caliber including an ultrasonic aspirator (10–12). The use of exoscopes helps minimize the obstruction due to the small corridor, and provides ergonomic surgical positioning for retractors placed at obtuse angles (10–12).

## Conclusions

A subset of metastatic brain tumors occurs in deep-seated locations. Accessing and resecting these lesions can be associated with significant morbidity because it involves large craniotomies, extensive white matter dissection, prolonged retraction, and risk of inadvertent tissue injury. As a result, only palliative treatment options are typically offered for these lesions including observation, needle biopsies, and/or radiation therapy. With the development of new surgical tools and techniques, minimally invasive techniques have allowed for the treatment of these lesions previously associated with significant morbidity. These techniques include laser interstitial thermal therapy and channel-based resections.

## Author contributions

LM-H played a role in manuscript preparation, manuscript revision, figure edits, and critical evaluation. KA-E played a role in manuscript preparation, manuscript revision, literature search. KC played a role in manuscript preparation, final approval, supervision.

### Conflict of interest statement

KC is a course lecturer for NICO Corporation. The remaining authors declare that the research was conducted in the absence of any commercial or financial relationships that could be construed as a potential conflict of interest.
